# 

**DOI:** 10.1192/bjb.2017.19

**Published:** 2018-02

**Authors:** Jennifer Parker

**Affiliations:** Core Psychiatry Trainee, Avon & Wiltshire Mental Health Partnership NHS Trust; email: jenniferparker@doctors.org.uk

Stigma remains pervasive in mental healthcare, despite efforts to challenge and quell it. Stigma begets more stigma, and a combination of discrimination, prejudice, misunderstanding and stereotyping can ensnare people with mental illnesses in a cycle of self-stigma.

Through a series of discursive essays derived from conversations with her partner, Jo McFarlane reflects on her decades-long experience of mental illness and the way in which she has been treated by clinicians and by society. She uses a ghetto as a harrowing metaphor for the isolation, vulnerability and feelings of entrapment which stigma has enforced upon her. As the metaphor suggests, her journey towards recovery and self-fulfilment is rife with challenges. However, patient-centred therapeutic relationships, the opportunity to volunteer, informal peer support, and the creation and dissemination of art have all been instrumental in allowing her to achieve her full potential as an individual and as a member of society. She rightly throws shade on the patriarchal role psychiatry has historically had, in which her sexuality and social choices were pathologised rather than embraced, reminding us of the importance of a holistic and open-minded approach to care.

What I found most engaging about the book was the way in which creative endeavour was presented by the author not simply as catharsis but as a fulcrum for self-discovery, allowing her to shift her identity ‘from patient to poet’ and rise above the stigma which once shackled her. Furthermore, she uses her writing to give a voice to others, the oppressed ‘ghettoised’ masses, thus showing the role of art in activism and advocacy.

This book serves as a reminder of the importance of recognising and tackling stigma, particularly self-stigma, which I intend to continue to reflect upon as I embark on a career in psychiatry myself. It is an accessible, thought-provoking and stimulating read with occasional space for humour, and I believe it would be a worthwhile read not only for those working in the field of psychiatry, but also for those using mental health services.

